# Feasibility Study of Dual Energy Radiographic Imaging for Target Localization in Radiotherapy for Lung Tumors

**DOI:** 10.1371/journal.pone.0108823

**Published:** 2014-09-30

**Authors:** Jie Huo, Xianfeng Zhu, Yang Dong, Zhiyong Yuan, Ping Wang, Xuemin Wang, Gang Wang, Xin-Hua Hu, Yuanming Feng

**Affiliations:** 1 Department of Biomedical Engineering, Tianjin University, Tianjin, China; 2 Department of Radiation Oncology, Tianjin Cancer Hospital, Tianjin, China; 3 Department of Physics, East Carolina University, Greenville, North Carolina, United States of America; 4 Department of Radiation Oncology, East Carolina University, Greenville, North Carolina, United States of America; National Cancer Center, Japan

## Abstract

**Purpose:**

Dual-energy (DE) radiographic imaging improves tissue discrimination by separating soft from hard tissues in the acquired images. This study was to establish a mathematic model of DE imaging based on intrinsic properties of tissues and quantitatively evaluate the feasibility of applying the DE imaging technique to tumor localization in radiotherapy.

**Methods:**

We investigated the dependence of DE image quality on the radiological equivalent path length (EPL) of tissues with two phantoms using a stereoscopic x-ray imaging unit. 10 lung cancer patients who underwent radiotherapy each with gold markers implanted in the tumor were enrolled in the study approved by the hospital's Ethics Committee. The displacements of the centroids of the delineated gross tumor volumes (GTVs) in the digitally reconstructed radiograph (DRR) and in the bone-canceled DE image were compared with the averaged displacements of the centroids of gold markers to evaluate the feasibility of using DE imaging for tumor localization.

**Results:**

The results of the phantom study indicated that the contrast-to-noise ratio (CNR) was linearly dependent on the difference of EPL and a mathematical model was established. The objects and backgrounds corresponding to ΔEPL less than 0.08 are visually indistinguishable in the bone-canceled DE image. The analysis of patient data showed that the tumor contrast in the bone-canceled images was improved significantly as compared with that in the original radiographic images and the accuracy of tumor localization using the DE imaging technique was comparable with that of using fiducial makers.

**Conclusion:**

It is feasible to apply the technique for tumor localization in radiotherapy.

## Introduction

As a method for tissue discrimination, dual energy (DE) imaging has been shown to be of good performance in thoracic [1], cardiac [2] and mammographic [3] imaging applications. In DE imaging the acquired images are combined to effectively separate an imaged object into distinct component images of specific tissue types or tissue-selection for generating high contrast images of targeted structures, which can be applied to improve tumor detection for diagnostic interpretation.

Planar kilovoltage (kV) imaging plays an important role in image guidance in radiation therapy (RT) systems, such as CyberKnife (Accuray, Inc., Sunnyvale, CA), ExacTrac (Brainlab AG, Feldkirchen, Germany) and others. It operates by acquiring two radiographs of the patient's anatomy in the treatment room at two different beam angles in real-time and comparing them with pre-generated digitally reconstructed radiographs (DRRs) from the computed tomography (CT) image data used in the RT planning. This procedure is designed to monitor the position variation of the patient's anatomy in the CT coordinate frame [4]. The projection of three dimensional (3D) structures into a two dimensional (2D) image can result in obscuration of the structure of interest such as a lung nodule by overlying structures such as the ribs, which has been identified as a major limiting factor in the detection of lung nodules in radiographs [5].

As DE imaging could provide tissue-selecting images by eliminating the overlying structures, it could bring potential benefits of improved tumor localization if DE imaging can be applied using the kV image guidance unit. Additionally, tumor localization without implanted metallic or radio frequency fiducials may eliminate a number of problems such as pneumothorax and hemorrhage [6]. Previous studies have explored the optimization of DE image acquisition parameters including kVp combinations [7], differential beam filtration and dose allocation [8] based on different image quality metrics such as contrast-to-noise ratio (CNR) [9], signal-to-noise ratio (SNR) [4], signal difference to noise ratio (SDNR) [10] and detectability index [11]. However, much more remains to be investigated quantitatively on the attenuation of x-ray as a function of the properties of the intervening tissues (such as atomic composition, mass density and thickness) in additional to the photon energy [12]. Enhanced discrimination of targeted structures such as tumor tissues through optimized DE imaging can only be achieved through a clear understanding of the effects of intrinsic tissue properties.

The purpose of this report was to quantify the effects of intrinsic tissue properties on tissue discrimination in DE images and to investigate the feasibility of applying the technique in radiotherapy image guidance systems such as the CyberKnife. We first derived the mathematical model for generating bone-canceled images. Then we conducted experimental studies with two phantoms to quantitatively analyze the influence of intrinsic tissue properties (represented by EPL) on the CNR of the image. Finally we applied the DE imaging technique to 10 lung cancer patients to evaluate the accuracy for tumor localization by comparing the results with that using the existing method of implanted fiducial markers.

## Materials and Methods

### 1. Modeling of bone-canceled image

DE imaging exploits differences in the photoelectric and Compton cross sections of different type of tissues in the object as x-ray photon energy varies [13]. Since the photoelectric absorption is dependent sensitively on atomic number, bony structures with high calcium concentration present different image contrast in the high energy (HE) radiograph from that in the low energy (LE) radiograph. Therefore, tissue-selecting image can be obtained by combining the two radiographs of different energies. A common algorithm for DE image reconstruction, derived from the Beer–Lambert law, is to apply a weighted log-subtraction scheme [1]. If we assume that an object consists of bone, lung tissue and other soft tissues, the transmitted intensities of x-ray beams at two different energies of HE and LE can be written as
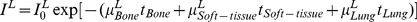
(1a)

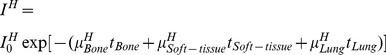
(1b)where *L* and *H* denote LE and HE, respectively, *I*_0_ and *I* are the intensities of incident and transmitted x-ray beams,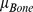, 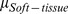, and 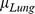 are the linear attenuation coefficients of bone, soft-tissue, and lung tissue respectively, and 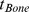, 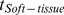, and 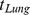 are the thicknesses of bone, soft-tissue, and lung tissue respectively. Because of the linear relationship with the transmitted intensities, the grey-level values of pixels in a HE or LE image can be expressed by Eq. (1) except a proportional constant.

A “bone-canceled” DE image can be calculated from a pair of HE and LE images as the following,
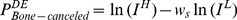
(2)where 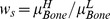 is the ratio of the bone attenuation coefficients to the HE beam and to the LE beam used as a weighting coefficient. In practice, because of the difference of imaging systems (such as difference in beam filtration and spectrum of energy, etc.), the calculated value of *w_s_* is not necessarily the ideal value for a specific DE imaging study. In this study, we set up *w*_s_ from 0.05 to 1 with a step size of 0.05 in the calculation of bone-canceled DE images and chose the optimized value to best eliminate the bony structures in the DE images. Substituting the *I^L^* and *I^H^* given by Eq. (1a) and (1b) into Eq. (2), one can derive the bone-canceled image as
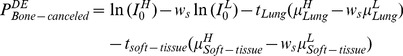
(3)


For the concerned energy range of diagnostic imaging, the x-ray interaction with tissues is dominated by photoelectric absorption and Compton scattering. Attenuation coefficient can therefore be decomposed into two components as given by the following approximate form [14],
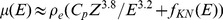
(4)where 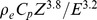 provides the photoelectric absorption coefficient with a fitting parameter of 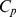 =  9.8×10^−24^, photon energy *E* in keV [14] and Z as the effective atomic number while the Klein-Nishina function 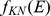 yields the electronic cross section of Compton scattering which depends only on *E* with 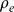 as the electron density. Combining Eq. (3) with Eq. (4) leads to Eq. (5).
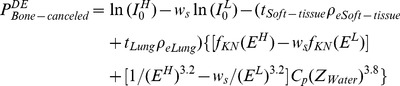
(5)


Here, we used 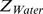 to replace 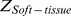 and 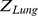 due to the following fact. Given the definition of effective atomic number as 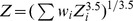, with 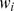 as the weight fraction of the element *i* of atomic number 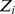 [15], the effective atomic number of soft-tissue and lung tissue can be found as 7.5666 and 7.5881, respectively, which are close to 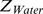 at 7.6843 [16]. To correlate the grey-level values of pixels in the bone-canceled image with the characteristics of the tissues that the x-ray photons transport through, a parameter of radiological equivalent path length (EPL) is used which is usually defined as the summed products of the thickness 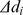 of a bone-excluding tissue component *i* and the ratio of electron density of the tissue component to that of water 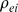 as given by Eq. (6).
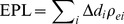
(6)


Hence, Eq. (5) can be simplified with EPL,
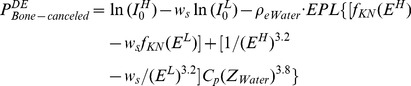
(7)where EPL accounts for the accumulated equivalent path length that x-ray photons pass through except the tissue to be canceled, i.e., bone.

The model expressed in Eq. (7) correlates the grey-level values of pixels in the bone-canceled image with the characteristics of bone-excluding tissues in terms of the EPL and the photon energies clearly. Therefore, the dependence of the image quality on the tissue properties expressed by EPL and image acquisition parameters can be quantitatively analyzed.

### 2. Evaluation of image quality

The visibility of targeted structures in a radiograph is largely dependent upon the absolute signal difference and the noise in the image, which can be related to the parameter CNR as defined below, 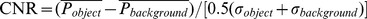
(8)where 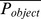 is the averaged grey-level values of pixels in an object region, and 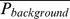 is the averaged grey-level values of pixels in an adjacent background region. 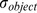 is the standard deviation (SD) of grey-level values in an object region, and 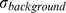 is the SD of grey-level values in an adjacent background region, which are calculated as the root-mean-square of the grey-level variance for all pixels in the regions respectively. CNR was used in this study for the quantitative evaluation of bone-canceled image quality.

### 3. Phantom study

**Figure 1:**
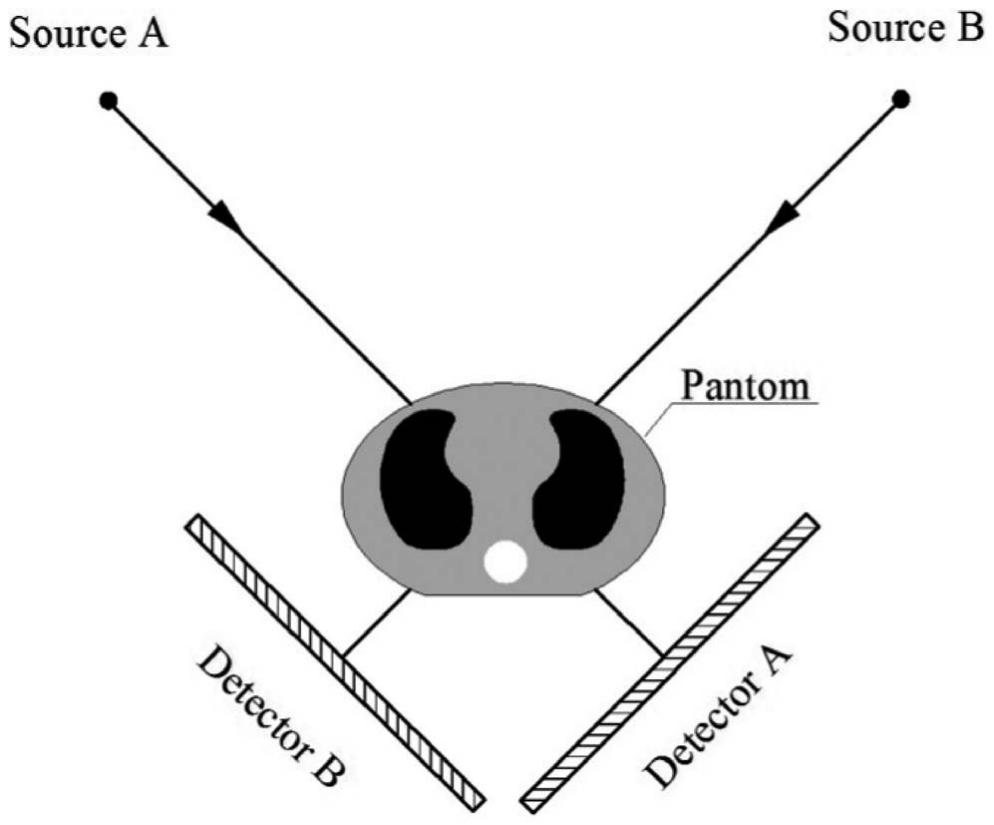
Geometric schematic of the radiographic imaging unit. The imaging system uses 2 diagnostic X-ray sources (Source A and Source B) mounted on the ceiling and paired with 2 flat panel detectors (Detector A and Detector B) with the same source-to-detector distance (SDD) of 3 m to acquire real-time digital radiographic images of the patient. The patient is imaged at 45 degree LAO (left anterior oblique) and RAO (right anterior oblique) angles to facilitate target localization in the 3D space.

We employed two phantoms to investigate the influence of the EPL on the DE image quality. HE and LE radiographs of the phantoms were acquired with the kV radiographic imaging unit of a CyberKnife system (G3, Version 7.1.1). The x-ray detector of the unit has a 20×20 cm^2^ field of view (FOV) with a 392 µm pixel size. The geometry of the imaging unit is shown in Figure 1.

Two elliptic cylinder phantoms, a chest phantom (Model 002LFC, CIRS, Norfolk, VA) and an Xsight Lung Tracking (XLT) phantom (Model 18023, CIRS), were used to assess the impacts of the tissue EPL on the quality of bone-canceled image. The first phantom has dimensions of 30×20×30 cm^3^ and simulates the structure of human chest consisting of three tissue equivalent components mimicking soft tissue (water), lung, and bone of which the relative electron densities are 1.002, 0.207, and 1.506, respectively. The second one has an elliptic cylinder configuration with dimensions of 30×20×18 cm^3^ and tissue-equivalent inserts mimicking cortical bone, lung, soft tissue, and a tumor, of which the relative electron densities are 1.782, 0.207, 1.002, and 1.028, respectively.

According to the published data [8], optimal DE image quality can be achieved with the beam energy combination of 120 kVp and 60 kVp for HE and LE images respectively. With this combination, the milliampere seconds (mAs) were set at 7.5 and 90 for the HE and LE images respectively in our phantom study for obtaining optimal image quality and avoiding overheating of the x-ray tubes during the image acquisition. The bone-canceled images of the chest phantom were obtained by combining the HE and LE images according to Eq. (2). In order to quantify the quality of bone-canceled images with CNRs, twelve ROIs of 12 pixels × 12 pixels in each bone-canceled image were identified with six as the object regions and six as the background regions. Each ROI was divided into 9 sub-areas for the calculation of mean value and SD of CNR. Then the CNRs were calculated using Eq. (8) to quantitatively analyze the impact of EPL. The EPL difference between an object region and a background region normalized by the averaged EPL level of the two regions (ΔEPL) was derived and the correlation between the CNR and ΔEPL was established and tested by evaluating the agreement of the calculated relationship between ΔEPL and CNR among the two different phantoms.

The EPL of each ROI is the averaged value of the region and was calculated according to the method described in the next section.

### 4. Calculation of EPL

EPL calculation was performed in the volumes of objects represented by their 3D CT image data sets. We used the ray tracing algorithm [17] to track the exact radiological path by propagating the incident x-ray photons through an object's 3D voxels. Following steps were used in the calculation of EPL. First, the 3D volume of a calibration phantom with known electron densities (Model 062, CIRS, Norfolk, VA) was reconstructed from the CT image data acquired with a Brilliance Big Bore CT simulator (Philips, Cleveland, OH) at 120 kVp and 400 mAs. This allowed us to build a look-up table to correlate the electron density of the object with the CT numbers in the 3D image sets acquired by the same scanner. The next step was to identify all voxels on the trajectories of x-ray photons propagating through a study phantom, the XLT or chest phantom, from source to the corresponding pixels of a flat panel detector placed behind the phantom. Once the voxels were identified we could retrieve the electron density values of these voxels from the look-up table based on their CT numbers and distinguish the bony structures at the same time. Finally, the values of EPL on the photon trajectories were summed by integrating along the path excluding bones since they were performed on the bone-canceled images.

### 5. Feasibility study with patient data

Image data from 10 patients with lung cancers who underwent SBRT with the CyberKnife system were used with the approval of the Ethics Committee of Tianjin Cancer Hospital (#2013-10) and the patients' written consents. Before including a patient in the study, tumor movement range due to respiratory motion was evaluated with fluoroscopic imaging first to minimize the effect of motion artifacts. Patients with tumor movement ranges of less than 12 mm in a respiration cycle were selected and then trained for holding their body still and exercising shallower breathing before the image acquisition. Radiographic images of HE and LE were obtained sequentially during the radiation delivery at the same phases of respiration which was guaranteed by the synchrony respiratory tracking system. We also chose to only include patients who had nodule size ≥ 10 mm in diameter as the identification of small nodules is still problematic for DE imaging [18]. Image data from 10 patients were selected. Each patient had at least three gold fiducial markers implanted before their CT simulation for treatment planning. And the maximum displacement of the tumors was checked with the markers to be ≤ 0.23 mm between the HE and LE images at the same phases of respiration.

The DRRs generated with the simulation CT image data for the image guidance of the treatment were used for comparison in the study, which carried the projected gross tumor volume (GTV) contours delineated in the CT images by an experienced radiation oncologist. HE and LE images (120 kVp, 100 mA, 75 ms and 60 kVp, 250 mA, 150 ms) were acquired with the image guidance unit before the start of treatment. Then bone-canceled images were obtained using the above discussed method and the GTVs were delineated in the bone-canceled images using an algorithm for automatically detecting pulmonary nodules in chest x-ray images [19]. The averaged displacement of the gold marker centroids between in the DRR and in the bone-canceled image was used as the reference for the GTV position variance analysis for each patient. The difference between the variation of the centroid of the delineated GTV in the DRR and in the bone-canceled image and the averaged variation of gold marker centroids in the two image sets was used to quantify the accuracy and evaluate the feasibility of the proposed DE imaging method for GTV localization. To quantitatively evaluate the improvement of tumor contrast, CNRs of the tumors in the radiographs at LE and HE, and in the bone-canceled DE images were calculated respectively using the same method as described in Section 2.3.

Statistical analysis was performed with GraphPad Prism 6 (GraphPad Software, Inc., La Jolla, CA). A 2-sided Student's t-test was used to compare the displacement between fiducial centroids and the GTV centroids, and evaluate the improvement of CNR of tumors with the DE imaging technique. A p-value of less than or equal to 0.05 was regarded as statistically significant.

## Results

### 1. Influence of ΔEPL on CNR

**Figure 2:**
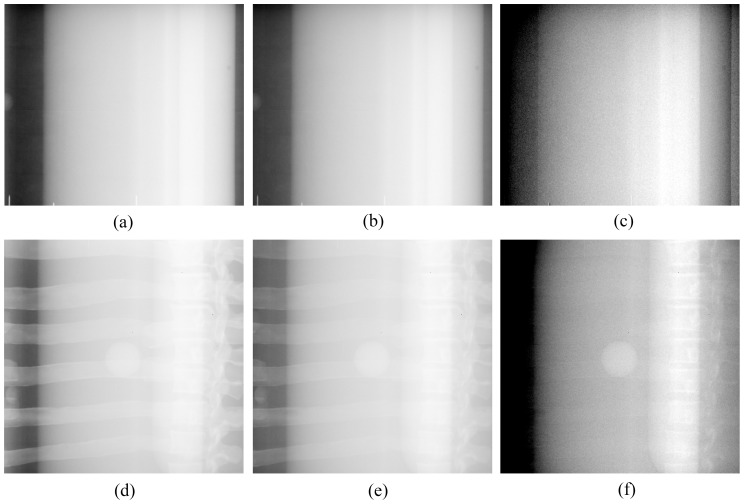
Radiographic images of chest phantom. (a), (b), and (c) are the images of chest phantom at 60 kVp, 120 kVp, and the bone-canceled image by combining (a) and (b), respectively. (d), (e), and (f) are the images of XLT phantom at 60 kVp, 120 kVp, and the bone-canceled image by combining (d) and (e), respectively.

**Figure 3:**
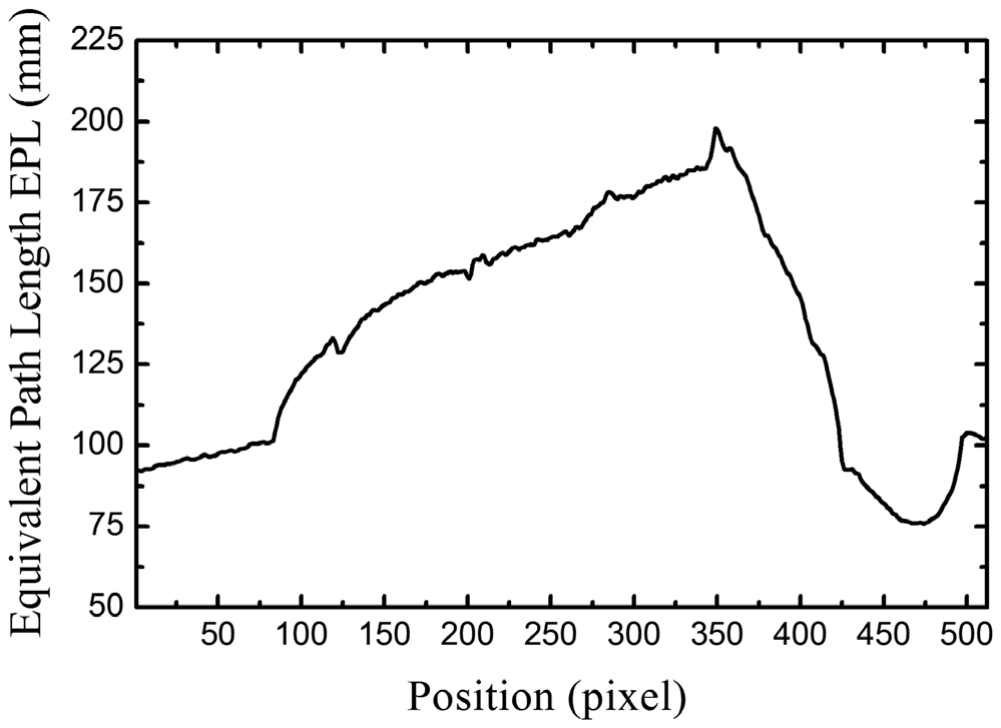
Averaged EPL per pixel column in the bone-canceled image of chest phantom.

**Figure 4:**
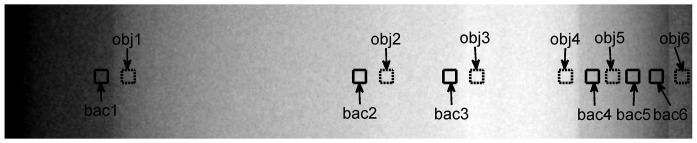
ROIs used in CNR-ΔEPL analysis of the bone-canceled image of chest phantom.

Figures 2(a) and 2(b) show the planar x-ray images of the chest phantom at 60 kVp and 120 kVp, respectively, and Figure 2 (c) is the bone-canceled image calculated from these two images. Figure 3 is the averaged EPL corresponding to every column in the image (512 columns) which was calculated according to the method described above. By comparing Figure 2(c) and Figure 3, it can be observed that the EPL is proportional to the grey-level value of the corresponding pixel, which corroborates with the model expressed in Eq. (7). Measurement of CNR in Figure 2(c) and data fitting using the linear-least-squares method showed that a linear relationship exists between CNR and ΔEPL. The result was evaluated with the parameter of coefficient of determination R^2^, which indicates how well measured data fit a statistical model and ranges from 0 to 1 with 1 indicating a perfect fit. The evaluation yielded a very high R^2^ value (0.99), which means that the CNR in bone-canceled image as a function of ΔEPL can be expressed by Eq. (9). Figure 4 plots the CNR against ΔEPL, the dots represent measured data and the solid line is the modeled result.
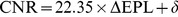
(9)


Because of noise, CNR can fluctuate in DE image for tissue trajectories with same EPL which is represented by δ in Eq. (9). Figure 2 (d) and (e) show the planar x-ray images of the XLT phantom acquired at 60 kVp and 120 kVp, respectively, and Figure 2(f) is the bone-canceled image. The CNR dependence on ΔEPL as expressed in Eq. (9) was also verified with the XLT phantom with the slope given by 23.29 and the difference was 4.2% between the two phantoms. Therefore, for the image guidance unit, the correlation of CNR with ΔEPL can be written as
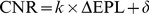
(10)where k is a system parameter with value around 22.8 and equal to the averaged value of the slope for the two phantoms.

**Figure 5:**
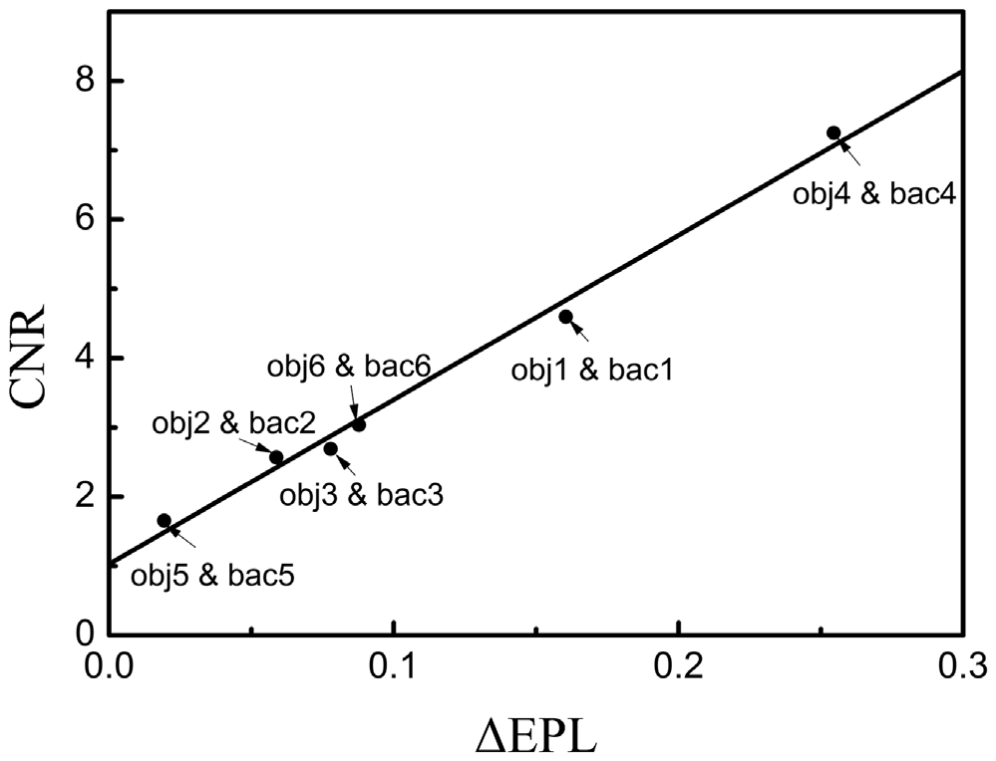
CNR versus ΔEPL in the bone-canceled image of chest phantom.

Figure 4 presents the bone-canceled image with ROIs of the chest phantom selected for the CNR calculation, and the corresponding CNR values are indicated in Figure 5. The squares in dash line are the object ROIs (marked as obj) while the ones in solid line are the background ROIs (marked as bac). By visual inspection, one can find that the visual differences between the ROI pairs of obj2 and bac2, obj3 and bac3, obj5 and bac5 cannot be observed; the visual differences between the ROI pairs of obj1 and bac1, obj4 and bac4, obj6 and bac6 can be observed. And the smallest observable difference in the image is between the pair of obj6 and bac6, of which the CNR is 2.57±0.34 (Mean ± SD). According to Figure 5, when CNR  =  2.91 (2.57+0.34), ΔEPL is 0.08, therefore, when ΔEPL is greater than 0.08, the difference between the object and the background in the bone-canceled image can be discriminated. In comparison, objects and backgrounds corresponding to ΔEPL less than 0.08 are visually indistinguishable.

### 2. Study with patient data

**Figure 6:**
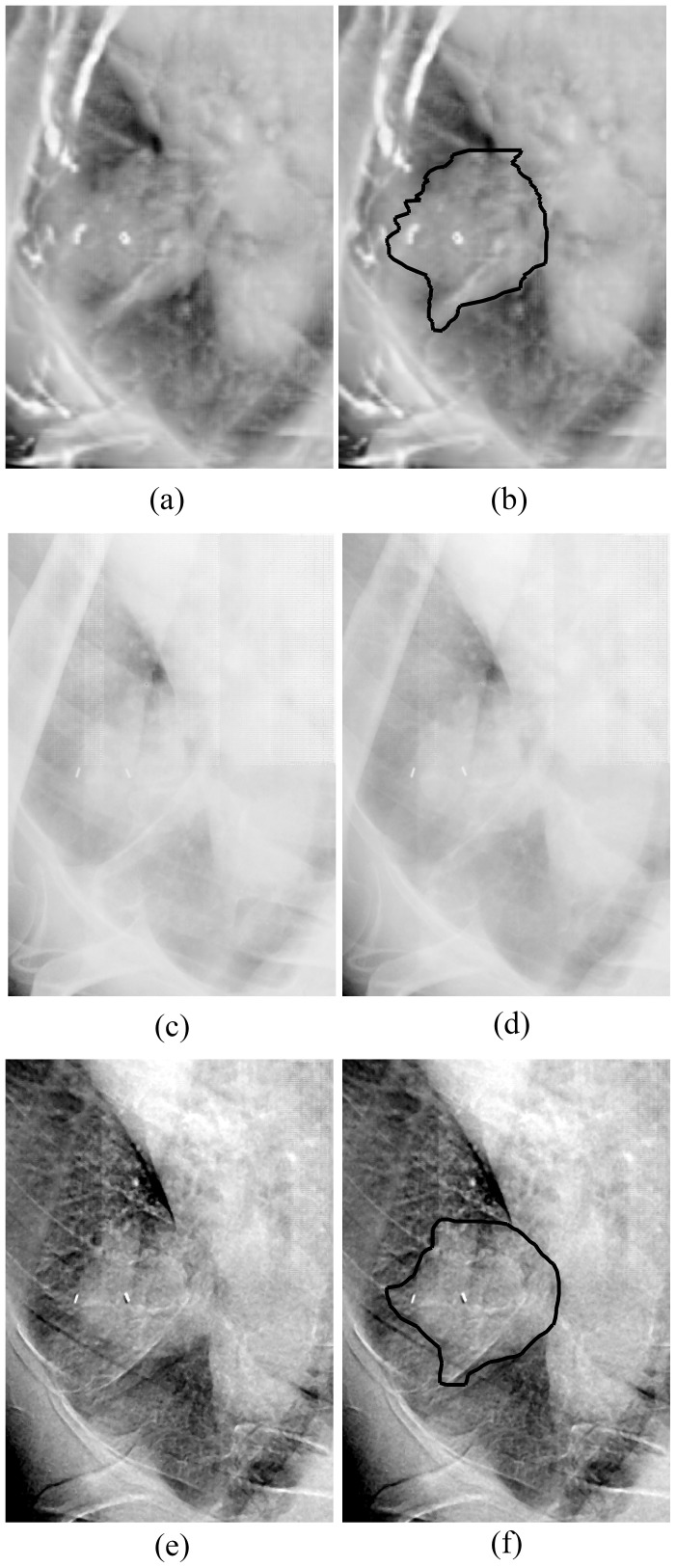
DRRs and radiographs of a patient. (a) DRR of a patient, (b) DRR with the projected GTV contour, (c) radiograph at LE, (d) radiograph at HE, (e) bone-canceled image by pairing two original radiographs, and (f) bone-canceled image with the GTV contour automatically segmented.

Figure 6 shows the DRRs, radiographic images and bone-canceled images of one of the patients. Calculation of CNR for the tumors showed that the mean value and SD of CNR of the 10 tumors was 5.22±2.96 in LE images, 7.43±3.33 in HE images and 8.29±3.56 in DE images, respectively. The p-value was 0.16 for the CNR in HE images versus in LE images, 0.01 for the CNR in DE images versus in LE images, and 0.03 for the CNR in DE images versus in HE images, respectively. This indicates that tumor contrast in the bone-canceled images is improved significantly as compared with those in the original planar radiographic images. The GTVs in the DRRs were projected from the planning RT data which were delineated and confirmed by an experienced radiation oncologist. And the contours in the radiographs were delineated with the automatic tumor detection algorithm as discussed above. Using the displacement of GTV centroid to represent the variation of tumor location, we compared the displacements of the fiducial centroids and the GTV centroids. The results are shown in Table 1. With a 2-sided Student's t-test, we found the p-value was 0.53 indicating that the variation measured by the two methods does not exhibit significant difference.

**Table 1:** Displacements of GTV centroids and fiducial markers.

Patient	D^a^ of GTV centroids (mm)	D^a^ of fiducials (mm)
1	4.22	4.22
2	6.36	4.36
3	5.63	3.05
4	6.67	6.06
5	9.37	7.82
6	6.09	6.63
7	1.11	0.40
8	15.03	11.94
9	3.11	3.15
10	2.81	2.50
Mean^b^	6.04	5.10
SD^c^	3.93	3.26

a D: Displacement.

b Mean: mean value.

c SD: standard deviation.

## Discussion

Dual-energy imaging technique holds promise to provide valuable information for improving target or tumor localization. However, its implementation as a practical clinical tool requires a clear understanding of the underlying mechanism for optimizing the image acquisition process and quality assurance. Specifically one needs to quantitatively characterize the intrinsic tissue properties to accurately assess the discrimination of targets. For this purpose, we have analyzed the DE imaging process and derived an expression for the image contrast CNR in terms of EPL which allows quantification of the target localization in a DE image.

Previously published studies focused mainly on the effect of phantom size on the quality of image. For example, Kappadath et al. employed a phantom consisting of different aluminum strips to simulate calcifications and breast-tissue-equivalent materials to evaluate the CNR in tissue-canceled DE images under the conditions of different strip thicknesses or glandular ratios [9]. The results showed that the CNR increases with increasing aluminum strip thickness and decreases with increasing glandular ratio in DE image, which is consistent with our results. However, the strip thickness used in that study differs from the realistic clinical situations. In contrast we utilized the anthropomorphic phantoms and the EPL to study the influence of intrinsic tissue properties with of improved clinical relevance. Although the qualitative relationship between EPL and CNR agrees with previous studies, the values of CNR obtained in our study differ because of the large variation in the imaging systems. As the image quality is influenced by detector performance such as the modulation transfer function (MTF), the noise-power spectrum (NPS), and the noise-equivalent quanta (NEQ), the image quality of different imaging system and CNR can vary widely. Therefore, it is necessary to calibrate the CNR-ΔEPL curve according to the specifications of each imaging system.

Finally, we would like to point out that the feasibility study with data of 10 patients has indicated that the tumor in the bone-canceled image as shown in Figure 6 can be easily seen with higher contrast than those in the planar radiographs. We expect that the DE imaging technique has the potential as a powerful tool for tumor localization. There are other advanced image processing methods and imaging techniques, such as rib suppression method [20] or MRI, which could provide similar image quality or superior soft tissue discrimination. But the feasibility for tumor localization in radiotherapy treatment with these advanced methods yet to be quantitatively evaluated.

## Conclusion

CNR is linearly dependant on ΔEPL in bone-canceled DE images and the relationship can be shown with a mathematical model as given by Eq. (10). The contrast for tumors in the bone-canceled images is improved significantly as compared with the one in the planar radiographic images and the accuracy of tumor localization using the DE imaging technique has been demonstrated to be comparable with that of using fiducial makers. Therefore, it is feasible to apply the technique for tumor localization in radiotherapy. Comprehensive clinical study with more patient data to model DE image quality with intrinsic tissue properties for tumor localization is ongoing and the results will be presented after completion of the study.
